# Risk of myocardial infarction and stroke following bloodstream infection: a population-based self-controlled case series

**DOI:** 10.1136/openhrt-2025-003241

**Published:** 2025-03-25

**Authors:** Jonathan Underwood, Nicola Reeve, Victoria Best, Ashley Akbari, Haroon Ahmed

**Affiliations:** 1 Infection and Immunity, Cardiff University, Cardiff, UK; 2 Infectious Diseases, Cardiff and Vale University Health Board, Cardiff, UK; 3 Division of Population Medicine, Cardiff University, Cardiff, UK; 4 Population Data Science, Swansea University, Swansea, UK

**Keywords:** Inflammation, STROKE, Myocardial Infarction

## Abstract

**Background:**

Cardiovascular disease (CVD) events triggered by inflammation are an underappreciated and poorly quantified cause of morbidity and mortality in patients with bloodstream infections (BSIs). We aimed to determine the risk of myocardial infarction (MI) and stroke after BSI.

**Methods:**

This self-controlled case series study was conducted within the Secure Anonymised Information Linkage Databank, containing anonymised population-scale electronic health record data for Wales, UK. We included adults with community-acquired BSI between 2010 and 2020. MI and stroke were determined from International Classification of Disease Version 10 coded admissions. Predefined risk periods after BSI were compared with the baseline period using pseudo-Poisson regression adjusted for age. Maximum C-reactive protein (CRP), a proxy for the magnitude of the inflammatory response, was determined within the first 7 days after BSI.

**Results:**

We identified 50 450 individuals with MI and 56 890 with stroke, of whom 1000 and 1290, respectively, also had at least one community-associated BSI. The risk of MI was most elevated in the first 1–7 days after BSI (adjusted incidence rate ratio (IRR) (95% CI): 9.67 (6.54 to 14.3)) and returned to baseline after 28 days. The risk was similarly elevated for stroke.

The largest magnitude of risk was observed for those with a maximal CRP>300 mg/L (MI IRR: 21.54 (9.57 to 48.52); stroke IRR: 6.94 (3.14 to 15.32)).

**Conclusion:**

BSI is associated with an increased risk of CVD events in the first 2 weeks after infection. Greater systemic inflammation was associated with a higher risk of CVD events and suggests targeting the inflammatory response caused by BSI warrants further study.

**WHAT IS ALREADY KNOWN ON THIS TOPIC**

- Previous studies using have shown an elevated risk of cardiovascular events following bloodstream infection (BSI), but interpretation is limited by study design and confounding.

**WHAT THIS STUDY ADDS**

- BSIs are clearly associated with a substantially increased risk of myocardial infarction and stroke in the first 2 weeks after infection. The risk increases with the magnitude of the inflammatory response, suggesting systemic inflammation plays a key mediating role.

**HOW THIS STUDY MIGHT AFFECT RESEARCH, PRACTICE OR POLICY**

- Our findings add to the growing body of evidence linking acute infections with elevated short-term cardiovascular risk. Future research should focus on exploring treatment strategies that target the inflammatory response in patients with BSIs, in addition to antimicrobial therapies.

## Introduction

 Bacteraemias, or bloodstream infections (BSIs), are common, life-threatening infections with a reported 30-day mortality ranging from 15% to 30%^1^,^3^ and are responsible for hundreds of thousands of deaths each year worldwide.^1^ Previous work from our group has shown that 3–7% of people with BSI actually die from ischaemic heart disease or cerebrovascular disease, depending on the infecting organism and timing of death.^4^

The relationship between BSI and cardiovascular disease (CVD) is multifactorial and is likely due to (1) endothelial dysfunction, atherosclerotic plaque destabilisation and thrombosis (eg, type 1 myocardial infarction (MI) provoked by inflammation-mediated activation of the coagulation cascade and platelets^5^) and (2) supply-demand imbalance (eg, type 2 MI provoked by sepsis/hypotension^6^).

To date, associations between acute infections and incident MI and stroke have been reported, most notably for influenza and community-acquired pneumonia.^7 8^ However, most studies used designs susceptible to bias and/or did not use laboratory-confirmed infections. More recent work used laboratory confirmation and study designs less susceptible to confounding (eg, self-controlled case series—SCCS) and found clear associations between respiratory tract infections and the risk of MI.^9^ For BSI, cohort data has shown an increased risk for MI or stroke within 30 days of BSI.^10 11^ Further evidence of the short-term risk of MI with *Staphylococcus aureus* BSI (SAB),^12^ using the SCCS design, which inherently controls for confounding better than standard observational studies.

Determining the risk and timing of CVD events after common causes of bacteraemia (particularly *Escherichia coli,* given its ubiquity and paucity of data about CVD risk) is important given the global burden of these infections^1^ and potentially preventable/treatable nature of CVD events. To test our hypothesis that BSI and its associated inflammation were associated with an increased risk of CVD events, we conducted a population-based study using the SCCS design and laboratory-confirmed BSI.

## Methods

### Study design and population

The SCCS only includes individuals who experience both exposure and outcome. Individuals act as their own control, with the risk of the outcome compared at different times within individuals, rather than between individuals. This inherently controls for within-subject time-invariant confounders, even if they are unmeasured or unknown. The incidence of the outcome is calculated for prespecified risk periods and compared with that of baseline periods. Assumptions of the SCCS are discussed in online supplemental table 1.

We used data from the Secure Anonymised Information Linkage (SAIL) Databank, the national trusted research environment for Wales, which holds ISO27001 certification. The SAIL Databank contains anonymised population-scale, individual-level linkable routinely collected data sources across a range of health and administrative data sources, including primary and secondary care from National Health Service (NHS) Wales. Details on the data sources can be found in online supplemental table 2. Eligible individuals were those who were Welsh residents during the observation period, aged between 30 and 100 years, with a first hospital admission for MI or stroke (recorded as a primary or secondary diagnosis in hospital data) between 1 April 2010 and 31 December 2020. Records with poor quality data linkage were excluded (online supplemental table 3). International Classification of Disease Version 10 (ICD-10) and Read codes used in the analyses are described in online supplemental table 4.

All study data were held within the SAIL Databank. Data access, research permissions and approvals were obtained from the SAIL independent Information Governance Review Panel (IGRP), project numbers 0923 and 0972. Only aggregated outputs were approved for release to ensure individuals were not identified (all counts in this paper are rounded to the nearest 10; counts less than 5 were suppressed and denoted as such). Analyses were undertaken in R V.4.1.3, using the SCCS package V.1.5.

### Patient and public involvement

We developed this research in collaboration with members of the Wales Centre for Primary and Emergency Care Research Service Users group and the SAIL consumer panel, including discussion of analysis plans, review of findings and plans for dissemination.

### Procedures

We used the SAIL Databank to access the following linked data: Patient Episode Database for Wales (PEDW), Welsh Longitudinal General Practice data, Welsh Results Reporting Service (WRRS) and Welsh Demographic Service Data set. PEDW data contains ICD-10 coded diagnoses for individuals admitted to any Welsh hospital and Welsh residents treated in English hospitals. WRRS data includes all tests requested from primary and secondary care NHS Wales organisations processed and analysed in NHS Wales laboratories. As the NHS in Wales provides virtually all healthcare in a nationally standardised healthcare system, routinely collected data is obtained and recorded similarly across the country.

Outcomes were acute MI or stroke. These were identified using ICD-10 codes from PEDW data on inpatient diagnoses (online supplemental table 4). Only the first MI or stroke in the observation period was included. For the primary analysis, the date of MI or stroke was defined as the hospital episode start date.

Exposures were all community-associated BSI during the observation period. All microbiological samples taken from NHS primary and secondary care services in Wales are processed in UK Accreditation Services accredited laboratories using standardised methodology with data stored in Public Health Wales’ datastore. Blood cultures that grew *E. coli, Klebsiella sp, Pseudomonas aeruginosa* and *S. aureus* from 1 April 2010 to 31 December 2020 were extracted from Public Health Wales’ data as previously described.^4^ Community-associated BSI were defined as having a blood culture collection date within admission date plus two calendar days and at least 28 days after any previous hospital admission period. The date of infection was defined as the blood culture collection date. Where more than one BSI was recorded on the same day, these were combined into a single event.

### Statistical analysis

SCCS analysis uses predefined risk periods and compares incidence in risk periods with incidence in baseline periods (online supplemental figure 1). We used a 90-day risk period, where day 0 was the date of the BSI. Individuals with more than one BSI had more than one corresponding risk period. Where risk periods overlapped, the later period took precedence and the earlier period was shortened. We included a day 0 risk period to allow for the situation where an individual had the BSI recorded on the same day as the MI or stroke, as it is difficult to ascertain which of the exposure and outcome came first, violating the principles of the SCCS method. Including a day 0 risk period accounts for this, allowing the incidence to be accurately calculated in the risk periods of interest.^13^ Baseline periods were all other times besides risk periods.

To account for event-dependence of exposures, we used pseudo-Poisson regression to estimate incidence rate ratios (IRRs) and 95% CIs based on sandwich variance estimates for the risk of acute MI or stroke in risk periods compared with baseline periods. The SCCS design inherently controls for time-invariant covariates such as sex. We adjusted each model for age, as age is associated with the incidence of BSI, MI and stroke.

In a secondary analysis, we repeated the primary analysis using hospital-associated BSIs as the exposure. Hospital-associated BSIs are defined as those where the sample was taken three or more calendar days after the hospital admission date or where there was a prior hospital admission period within the previous 28 days. To assess the robustness of our findings, we undertook several predefined sensitivity analyses including using troponin >99th centile and troponin>10 × >99th centile to improve granularity on MI timing (details in online supplemental table 5).

## Results

We identified 50 450 individuals with an ICD-10 code for MI and 56 890 with an ICD-10 code for stroke (online supplemental figure 2). Of these, 1000 (2.0%) and 1290 (2.3%) individuals respectively also had at least one community-associated BSI during their observation period and were included in the primary analysis. People with MI were 57% male with a median (IQR) age of 77 years (69–84). People with stroke were 50% male with median age of 78 years (69–84) (tables1  2).

**Table 1:** Baseline characteristics of people with myocardial infarction (MI) and bloodstream infection (BSI) by acquisition

Myocardial infarction (MI)			
Variable	Community-associated BSI (n=1000)	Hospital-associated BSI (n=1210)	P value
Age at MI (median, IQR)	77 (69–84)	76 (68–83)	0.03
Gender (n, %)			
Female	430 (43)	470 (39)	0.08
Male	570 (57)	730 (61)	
Ethnic group (n, %)			
Asian	<10 (1)	<10 (1)	0.81
Black	<10 (0)	<10 (0)	
Mixed	<10 (0)	<10 (0)	
Other	<10 (0)	<10 (0)	
White	580 (58)	690 (57)	
Missing	410 (41)	500 (41)	
WIMD 2019 quintile (n, %)			
1. Most deprived	200 (20)	270 (22)	0.4
2.	240 (24)	250 (21)	
3.	200 (19)	260 (21)	
4.	190 (19)	220 (18)	
5. Least deprived	170 (17)	210 (17)	
e-Frailty (n, %)			
Fit	240 (24)	360 (30)	0.005
Mild	310 (31)	370 (31)	
Moderate	300 (30)	310 (26)	
Severe	150 (15)	150 (13)	
Hypertension (n, %)	630 (63)	700 (58)	0.02
Diabetes (n, %)	340 (33)	400 (33)	0.81
Cardiovascular disease (n, %)	520 (52)	590 (49)	0.26
Chronic kidney disease (n, %)	340 (34)	380 (32)	0.38
COPD (n, %)	160 (16)	170 (14)	0.26
Aspirin (n, %)	590 (59)	660 (55)	0.05
Antihypertensives (exc. beta-blockers) (n, %)	720 (71)	830 (68)	0.14
Beta-blockers (n, %)	510 (51)	570 (48)	0.14
Statins (n, %)	640 (64)	720 (60)	0.05
P2Y12 inhibitor (n, %)	240 (24)	260 (21)	0.13
Smoking status (n, %)			
Ex-smoker	480 (48)	550 (45)	0.40
Never smoker	190 (19)	220 (18)	
Current smoker	160 (16)	190 (16)	
Unclear	50 (5)	70 (6)	
Missing	130 (13)	180 (15)	
BSI organism (n, %)			
*E. coli*	780 (71)	740 (55)	<0.001
*Klebsiella sp*	90 (9)	170 (12)	
Polymicrobial	20 (2)	30 (2)	
*P. aeruginosa*	30 (2)	60 (4)	
*S. aureus*	180 (16)	360 (27)	
Peak CRP mg/L (mean, SD)	207.03 (119.05)	197.19 (109.75)	0.04

Counts rounded to the nearest 10.

BSIBloodstream infectionCOPDchronic obstructive pulmonary diseaseCRPC-reactive protein*E. coliEscherichia coliP. aeruginosaPseudomonas aeruginosaS. aureusStaphylococcus aureus*WIMDWelsh Index of Multiple Deprivation

**Table 2:** Baseline characteristics of people with stroke and bloodstream infection (BSI) by acquisition

Stroke			
Variable	Community-associated BSI (n=1290)	Hospital-associated BSI (n=1500)	P value
Age at stroke (median, IQR)	78 (69–84)	77 (68–84)	0.14
Gender (n, %)			
Female	650 (50)	620 (42)	<0.001
Male	640 (50)	870 (58)	
Ethnic group (n, %)			
Asian	<10 (1)	<10 (1)	0.63
Black	<10 (0)	<10 (0)	
Mixed	<10 (0)	<10 (0)	
Other	<10 (0)	<10 (1)	
White	750 (98)	890 (98)	
Missing	520 (40)	590 (39)	
WIMD 2019 quintile (n, %)			
1. Most deprived	280 (22)	320 (21)	0.5
2.	310 (24)	320 (21)	
3.	240 (19)	310 (20)	
4.	230 (18)	290 (19)	
5. Least deprived	230 (18)	260 (18)	
e-Frailty (n, %)			
Fit	340 (26)	440 (30)	0.09
Mild	440 (34)	450 (30)	
Moderate	340 (26)	390 (26)	
Severe	170 (13)	220 (15)	
Hypertension (n, %)	760 (59)	910 (61)	0.42
Diabetes (n, %)	380 (29)	420 (28)	0.56
Cardiovascular disease (n, %)	600 (47)	700 (47)	0.95
Chronic kidney disease (n, %)	360 (28)	440 (29)	0.45
Atrial fibrillation (n, %)	150 (15)	170 (14)	0.26
COPD (n, %)	160 (12)	180 (12)	0.83
Aspirin (n, %)	680 (53)	810 (54)	0.58
Antihypertensives (exc. beta-blockers) (n, %)	890 (69)	1040 (69)	0.96
Beta-blockers (n, %)	580 (45)	710 (48)	0.18
Statins (n, %)	750 (58)	850 (56)	0.40
P2Y12 inhibitor (n, %)	220 (17)	240 (16)	0.84
Smoking Status (n, %)			
Ex-smoker	570 (50)	630 (49)	0.37
Never smoker	300 (26)	340 (26)	
Current smoker	200 (18)	240 (19)	
Unclear	60 (5)	90 (7)	
Missing	160 (13)	190 (13)	
BSI organism (n, %)			
*E. coli*	900 (65)	900 (55)	<0.001
*Klebsiella sp*	110 (8)	200 (12)	
Polymicrobial	30 (2)	30 (2)	
*P. aeruginosa*	30 (2)	70 (4)	
*S. aureus*	300 (22)	450 (27)	
Peak CRP mg/L (mean, SD)	215 (120)	195 (110)	<0.001

Counts rounded to the nearest 10.

COPDchronic obstructive pulmonary diseaseCRPC-reactive protein*E. coliEscherichia coli*MImyocardial infarction*P. aeruginosaPseudomonas aeruginosaS. aureusStaphylococcus aureus*WIMDWelsh Index of Multiple Deprivation

### Risk of MI

**Figure 1:**
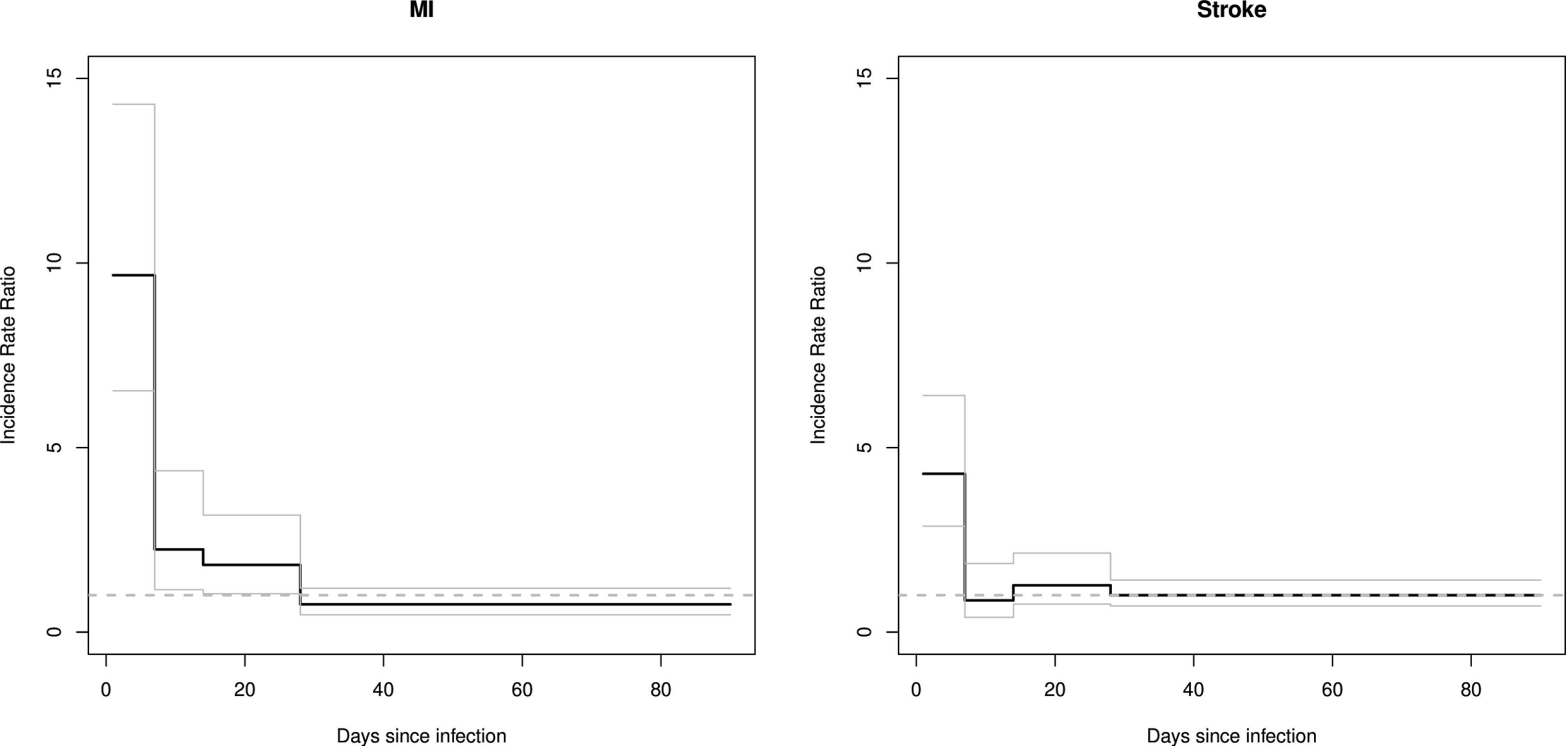
Incidence rate ratios for myocardial infarction and stroke after community-associated bloodstream infection (primary analysis).

Of 1000 identified MIs, 90 occurred during the risk periods of interest and 820 occurred during baseline time. The remaining 90 MIs occurred on day 0, which were excluded from further analysis due to issues ascertaining the temporality of exposure and event. Total observation time during risk periods was 82 937 days, and during baseline time was 2 927 115 days. The relative incidence of MI during days 1–7 following a community-associated BSI was 9.67 (95% CI 6.54 to 14.3). The risk declined over time and returned to baseline after 28 days (table 3 and figure 1).

**Table 3:** Crude and age-adjusted incidence rate ratio (IRR) for first myocardial infarction and first after community-associated bloodstream infection

Time period	MI				Stroke			
	No events*	Total Obs time (days)	Crude IRR (95% CI)	Age-adjusted IRR (95% CI)	No events*	Total Obs time (days)	Crude IRR (95% CI)	Age-adjusted IRR (95% CI)
Baseline	820	2 927 115	1	1	1080	3 623 612	1	1
1–7 days	50	7225	8.22 (5.57 to 12.13)	9.67 (6.54 to 14.30)	50	9214	3.48 (2.34 to 5.17)	4.29 (2.87 to 6.41)
8–14 days	10	6976	1.91 (0.98 to 3.72)	2.24 (1.15 to 4.37)	10	8749	0.70 (0.33 to 1.51)	0.86 (0.40 to 1.86)
15–28 days	10	13 437	1.56 (0.89 to 2.71)	1.82 (1.04 to 3.17)	20	16 751	1.04 (0.62 to 1.74)	1.27 (0.76 to 2.14)
29–90 days	20	55 299	0.64 (0.41 to 1.02)	0.75 (0.47 to 1.19)	50	68 247	0.83 (0.59 to 1.16)	1.00 (0.71 to 1.41)

* rRounded to the nearest 10.

MI, myocardial infarctionObsobservation

### Risk of stroke

Of 1290 identified strokes, 130 occurred during the risk periods of interest and 1080 occurred during baseline time. The remaining 80 strokes occurred on day 0. Total observation time during risk periods was 1 02 961 days and during baseline time 3 623 612 days. Similar to MI, the risk of stroke was elevated in the first 1–7 days after infection (adjusted IRR 4.29 (95% CI 2.87 to 6.41)). There was no statistically significant increase in risk in the 8–90 days after infection (table 3 and figure 1).

### Secondary analyses

**Figure 2:**
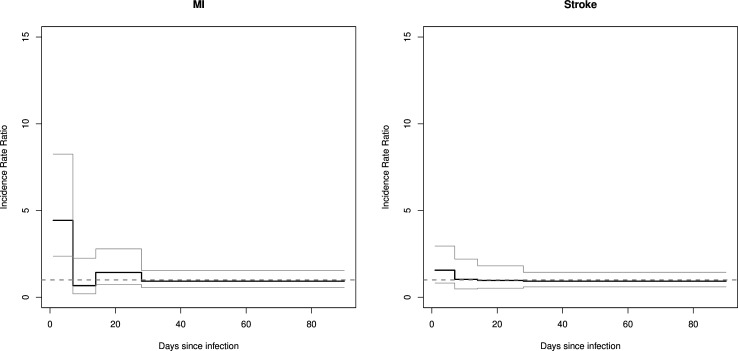
Incidence rate ratios for myocardial infarction and stroke after hospital-associated bloodstream infection (secondary analysis). MI, myocardial infarction.

Hospital-associated BSI were associated with similar, although attenuated, increased risks of CVD (table 4 and table 5). The relative incidence of MI in the first 7 days was 4.43 (95% CI 2.37 to 8.25), and of stroke was 1.56 (95% CI 0.82 to 2.95) (figure 2). Stroke risk was much higher for infection with *S. aureus* compared with *E. coli* (13.88 (6.78 to 28.41) vs 1.55 (0.76 to 3.14)), respectively, for risk up to 7 days (table 3 and online supplemental figure 3). The effect of gender on the risk of MI and stroke was not statistically significant (table 5).

**Table 4:** Crude and age-adjusted incidence rate ratio (IRR) for first myocardial infarction and first stroke after hospital-associated bloodstream infection

Time period	MI				Stroke			
	No events*	Total Obs time (days)	Crude IRR (95% CI)	Age-adjusted IRR (95% CI)	No events*	Total Obs time (days)	Crude IRR (95% CI)	Age-adjusted IRR (95% CI)
Baseline	1120	3 132 533	1	1	1380	3 816 191	1	1
1–7 days	20	8895	3.58 (1.94 to 6.62)	4.43 (2.37 to 8.25)	20	10 878	1.24 (0.66 to 2.31)	1.56 (0.82 to 2.95)
8–14 days	<5	8300†	–‡	0.67 (0.20 to 2.25)	10	10 121	0.82 (0.39 to 1.73)	1.03 (0.48 to 2.20)
15–28 days	10	15 036	1.18 (0.61 to 2.29)	1.43 (0.73 to 2.79)	20	18 583	0.78 (0.42 to 1.44)	0.97 (0.52 to 1.81)
29–90 days	30	56 295	0.79 (0.49 to 1.29)	0.93 (0.56 to 1.54)	40	69 581	0.76 (0.50 to 1.15)	0.93 (0.60 to 1.44)

* rRounded to the nearest 10.

† rRounded to the nearest 100.

‡ Excluded for disclosure reasons.

MI, myocardial infarctionObsobservation

**Table 5:** Age-adjusted incidence rate ratio (IRR) for myocardial infarction and stroke in the first 7 days after community-associated bloodstream infection—subgroup and sensitivity analyses

Sensitivity analyses(0–7 days)	MI		Stroke	
	No events*	Adjusted IRR (95% CI)	No events*	Adjusted IRR (95% CI)
Exclude subarachnoid haemorrhage	–	–	1260	4.35 (2.88 to 6.57)
Cerebral infarction and unspecified stroke only	–	–	1140	4.72 (3.07 to 7.26)
Additional 91–180 day risk period	–	–	1290	4.21 (2.77 to 6.41)
Troponin date as event date	1010	8.24 (5.46 to 12.43)	–	–
Troponin results in the 10×99th centile	1010	9.64 (6.49 to 14.32)	–	–
Exclude those who died within 30 days of the event	890	15.18 (10.24 to 22.5)	1060	5.89 (3.57 to 9.71)
Subgroups				
CRP concentration (mg/L)				
0–99	190	4.76 (1.73 to 13.11)	210	1.82 (0.41 to 8.04)
100–199	260	9.64 (4.76 to 19.52)	340	1.86 (0.69 to 4.97)
200–299	250	8.90 (4.19 to 18.89)	330	4.16 (1.79 to 9.69)
300+	210	21.54 (9.57 to 48.52)	280	6.94 (3.14 to 15.32)
Organism				
* E.coli*	720	8.77 (5.51 to 13.95)	850	1.55 (0.76 to 3.14)
* S. aureus*	160	10.34 (3.83 to 27.93)	260	13.88 (6.78 to 28.41)
Gender				
Male	580	10.81 (6.40 to 18.25)	640	5.04 (2.85 to 8.90)
Female	440	9.03 (5.00 to 16.3)	640	3.57 (2.00 to 6.36)
Statins				
Statins	650	7.75 (4.65 to 12.94)	770	4.01 (2.37 to 6.77)
No statins	350	14.21 (7.58 to 26.62)	540	5.36 (2.91 to 9.87)
Aspirin				
Aspirin	600	7.57 (4.34 to 13.2)	690	3.45 (1.99 to 5.97)
No aspirin	410	13.49 (7.75 to 23.48)	610	5.59 (3.11 to 10.04)

* Total number of events included in analysis, rounded to the nearest 10.

CRPC-reactive protein*E. coliEscherichia coli*MI, myocardial infarction

The risk of both MI and stroke was higher for individuals with higher C-reactive protein (CRP) concentrations (table 5 and online supplemental figure 4). The relative incidence of MI in the first 7 days increased from 4.76 (1.73 to 13.1) with a peak CRP of<100 mg/L to 21.54 (9.57 to 48.5) for those with CRP>300 mg/L. Numerically lower risk was observed for both aspirin and statin use, particularly for MI, but CIs were wide and overlapped (table 5 and online supplemental figure 5). The relative incidence of MI in the first 7 days was 7.75 (4.65 to 12.9) for people prescribed statins compared with 14.2 (7.58 to 26.6) in those who were not.

The results were robust to multiple changes of definitions and assumptions in the sensitivity analyses (table 5).

## Discussion

In this population-based study, we found community-associated BSI to be associated with incident MI and stroke. Risk was highest in the first 7 days after BSI and largely normalised 28-days after infection. Risk was clearly associated with the magnitude of the inflammatory response, whereby patients with the highest CRP concentrations were at the greatest risk of CVD events. Intriguingly, our results also suggest a possible protective effect of aspirin and statins. These findings were robust to a range of sensitivity analyses.

These findings are consistent with earlier studies reporting an increased risk of MI and stroke following BSI.^10^,^12^ Building on this work, we show that risk is mediated by the magnitude of the inflammatory response to BSI as well as infecting organism. The magnitude of associations we report is smaller than the previous largest study of community-associated BSI. Dalager-Pederson *et al*^10^ reported an adjusted relative risk of 17.7 for MI and 25.8 for stroke in the first 30-days after BSI. However, their use of a cohort design and choice of control groups with minimal adjustment risks significant residual confounding, therefore potentially inflating estimates of risk. The only previous study using SCCS of SAB by Corrales-Medina *et al*^12^ reported an IRR for MI of 35.3 and 7.9 during the first 2 and 14 days after SAB respectively. The discrepancy with our findings may be due to our inclusion of a separate day 0 risk period to account for the uncertainty about which event (ie, BSI or MI/stroke) happened first. Including a separate day 0 risk period is recommended to avoid bias and inflation of effect estimates in SCCS and is discussed in more detail in the methods.^14^ Furthermore, in this study, only 11 MIs occurred during the risk period (82% of which occurred within 2 days), which limits the robustness of these findings.

The discrepancy in risk of stroke between *S. aureus* and *E. coli* BSI is likely explained by differential rates of endocarditis. Endocarditis is a common cause of community-associated SAB and has an established risk of stroke due to embolism from left-sided valvular infection. We did not have echocardiographic or coding data available to accurately ascertain the incidence of endocarditis in our cohort. However, previous work using case-control designs has shown similar increased risk of stroke with SAB, with the highest risks reported in patients with endocarditis (adjusted RR: 117, specific BSI organisms unclear).^10 11^

The relationship between hospital-associated BSI and CVD events was less clear. Elevated baseline risk in already hospitalised patients due to comorbidities in combination with fewer events may limit the power of our study.

The mechanism of BSI-associated cardiovascular events is likely multifactorial. Inflammation triggered by infection leads in some to atherosclerotic plaque disruption, endothelial injury and increased risk of thrombosis through activation of the coagulation system and platelets.^5 7^ This is compounded by haemodynamic changes and mitochondrial dysfunction that occur during severe infection, causing macrocirculatory and microcirculatory dysfunction leading to a mismatch between oxygen delivery and consumption.^15^ We clearly demonstrate a link between the magnitude of the inflammatory response and risk of CVD events. Patients with a CRP exceeding 300 mg/L had over four times the risk of MI and three times the risk of stroke compared with patients with CRP<100 mg/L. The magnitude of the inflammatory response in BSI is complicated and influenced by age, comorbidities, pathogen virulence, host-pathogen interaction and treatment factors. The smaller effect sizes previously reported for viral infections compared with BSI are likely explained by greater systemic inflammation precipitated by bacterial compared with viral infection. Together, these findings strengthen the hypothesis that infection-associated inflammatory events are proportional to the magnitude of the inflammatory response.

We did not have detailed data regarding symptoms before presentation or treatment, and so we cannot accurately determine which factor(s) are most important in determining the magnitude of systemic inflammation. However, given previous associations with mortality, this is clearly an area of research that needs further exploration.^4^ Earlier and more effective antimicrobial treatment will likely attenuate the inflammatory response; however, it is unclear if antibiotics given earlier in the patient pathway, such as prehospital, make much difference to outcomes compared with standard of care.^14^ Therefore, trials of immunomodulatory therapies targeting the host response to BSI and other severe infections in combination with the usual pathogen-directed therapy (ie, antibiotics) are warranted. Anticoagulation, antiplatelet drugs and statins should also be considered for trials. Our data suggest a possible protective effect of these drugs, particularly when considering these are prescribed to individuals with the highest CVD risk who would be expected to be most at-risk following BSI. However, in patients hospitalised with COVID, clinical trials of aspirin and simvastatin have been disappointing,^16 17^ and trials of therapeutic anticoagulation have been mixed.^18^ However, a recent meta-analysis of trials of statins in patients with COVID reported reduced mortality, suggesting some promise for this approach.^19^ These contrast with the clear benefits seen of immunomodulatory therapies such as corticosteroids and IL-6 receptor antagonists, suggesting immunomodulatory approaches may be preferred.^20 21^ Furthermore, over half of the patients included here were already taking antiplatelet drugs (with a similar number taking a statin), which limits eligibility for an antiplatelet trial as well as increasing the risk of major bleeding events if anticoagulants were given concurrently.

Our study provides useful data to design and power treatment trials, including CRP thresholds to guide participant enrichment to reduce heterogeneity and improve sensitivity, and suggests that CVD events should be collected as important secondary endpoints in BSI clinical trials. Furthermore, our data should prompt extra vigilance for CVD events in patients with BSI, particularly those with comorbidities and very elevated CRP concentrations, as they are likely to be at the highest risk of adverse outcomes.

Key strengths of our study are the use of laboratory-confirmed BSI to accurately confirm culprit organism and timing of specimen collection combined with the use of the SCCS methodology. This study design eliminates residual time-invariant confounding between people who do and do not have BSI, which is inherent in cohort studies. Furthermore, use of troponin results to pinpoint timing of MI and multiple sensitivity analyses make our findings robust and very unlikely to be due to chance. Excluding day 0 risk avoids reverse causation. In reality, the infection that leads to a positive blood culture and increased risk of CVD events will have been developing for days/weeks. This makes day 0 reverse causation biologically implausible and likely leads to an underestimation of CVD risk following BSI. However, we included a separate day 0 risk period to reduce bias and inflation of effect estimates. Furthermore, from a pragmatic perspective, it would be very difficult to design trials or give treatment to mitigate risk in this period before BSI diagnosis is confirmed, as it typically takes 24–48 hours to isolate and identify the culprit organism. The major limitation of our study is the reliance on clinical coding for diagnoses of MI and stroke with potential misclassification without independent assessment of clinical histories, ECG and neuroimaging data to confirm these diagnoses. The use of routinely collected troponin results and multiple sensitivity analyses mitigate this for MI, but similar blood-based biomarkers are not routinely used for stroke, and so an analogous approach could not be undertaken. Therefore, these data should be treated with more caution. Furthermore, the lack of physiological data or more detailed coding did not allow the distinction between type 1 and type 2 MI. However, the association with ischaemic stroke and that these were clinically coded diagnoses suggest a large proportion were type 1 MIs and all were of clinical significance (and not just a troponin leak associated with sepsis). Similarly, the lack of ECG data makes it impossible to determine what proportion of ischaemic strokes are due to new-onset atrial fibrillation precipitated by BSI. Further mechanistic work to delineate the precise pathophysiology of the associations we report are outside the scope of this study and would require prospective data collection. Detailed in-patient treatment data were also not available, so we cannot fully ascertain if antiplatelet and statin medications offer any protection for CVD events or if patients not taking antiplatelet drugs were anticoagulated.

In conclusion, we found compelling evidence of an increased risk of MI and stroke in the first 2 weeks following BSI, which was clearly associated with the magnitude of the inflammatory response. Our findings contribute to the growing body of knowledge of infection-associated CVD events and suggest that targeting the host response to infection may be a fruitful area for future research and improving patient outcomes.

## supplementary material

10.1136/openhrt-2025-003241 online supplemental file 1

## Data Availability

Data may be obtained from a third party and are not publicly available.
