# Supplementary material for: Risk of myocardial infarction and stroke following bloodstream infection: a population-based self-controlled case series
Source: Open Heart. 2025 Mar 25;12(1):e003241. doi: 10.1136/openhrt-2025-003241 (PMC11938248; doi:10.1136/openhrt-2025-003241)
Supplement: online supplemental file 1 [file openhrt-12-1-s001.doc]

# Myocardial infarction and stroke associated with bloodstream infection – a population-based self-controlled case series.

# Supplementary Material

# Contents

[Supplementary Table 1. Model assumptions and solutions to violations of those assumptions 2](#__RefHeading___Toc181874728)

[Supplementary Table 2. Data sources 2](#__RefHeading___Toc181874729)

[Supplementary Table 3. Good data matching is generally considered to be anonymised linkage field (ALF) status code of 1, 4 or 39. 3](#__RefHeading___Toc181874730)

[Supplementary Table 4. ICD-10 codes for acute myocardial infarction and stroke to identify potential cases 4](#__RefHeading___Toc181874731)

[Supplementary Table 5.Sensitivity and subgroup analyses. 5](#__RefHeading___Toc181874732)

[Supplementary Figure 1. Diagrammatic representation of observation time for an individual in the proposed Self-Controlled Case Series design 6](#__RefHeading___Toc181874733)

[Supplementary Figure 2 Selection of myocardial infarction (MI) and stroke cases. 7](#__RefHeading___Toc181874734)

[Supplementary Figure 3. Incidence rate ratios for myocardial infarction and stroke by organism. 8](#__RefHeading___Toc181874735)

[Supplementary Figure 4. Incidence rate ratios for myocardial infarction and stroke by peak C-reactive protein concentration. 8](#__RefHeading___Toc181874736)

[Supplementary Figure 5. Incidence rate ratios for myocardial infarction and stroke by aspirin and statin use. 9](#__RefHeading___Toc181874737)

The SCCS method makes several assumptions that need to be met in order to obtain valid and unbiased estimates. In practice, these assumptions are not always met. There are model extensions which provide solutions to violations of these assumptions under certain circumstances.^1^The model assumptions, how they apply to our study, and the solutions to violations of the assumptions are given in Supplementary Table 1.

## *Supplementary Table 1. Model assumptions and solutions to violations of those assumptions*

| **Assumption** | **How the assumption applies to this study** | **Solution** | **Example of use of the solution in the literature** |
| --- | --- | --- | --- |
| **Subsequent exposures should not be affected by previous events.** | We might see a temporary increase in BSI subsequent to an MI or stroke event, which would bias estimates towards the null. However, these would not be classed as community BSI so should not affect outcomes. It is conceivable that MI/stroke would affect subsequent risk of BSI in the future however the numbers with repeat BSI are few. | Apply a pre-risk period. | Gibson et al. studied the association between prescription drugs and road traffic accidents. As some drugs may be used to treat anxiety or pain caused by the crash, a 4-week pre-exposure period was included.^2^ |
|  | As both MI and stroke have relatively high death rates, the length of the observation period is dependent on events, and no further exposures are possible after death. | Conduct a sensitivity analysis that repeats the analysis, excluding individuals who died within 30 days of the event. | Langan et al. studied the risk of stroke following herpes zoster. They conducted a sensitivity analysis excluding individuals who died within 90 days of stroke.^3^ |
| **Event rates are constant within defined periods** | MI and stroke are more common in older individuals and may be affected by seasonal changes. | Control for age and season effects. | Grave et al. studied the association between seasonal influenza vaccination and Guillan Barre syndrome. They adjusted for calendar month, as the vaccinations are seasonal by design.^4^  In a study of the association between chickenpox and stroke, Thomas et al. adjusted for age in 5-year age bands.^5^ |
| **Events are independently recurrent or rare.** | MI and stroke are not independent: once an individual has a first event, they are more likely to have a second. | Study first events only. | Langan et al. began the observation period 12 months into follow-up time to ensure first stroke events had been correctly identified.^3^ |

## Supplementary Table 2. Data sources

| **Dataset** | **Description** | **Data Coverage and Representativeness** |
| --- | --- | --- |
| Patient Episode Database for Wales (PEDW)^6^ | The PEDW data contains International Classification of Disease Version 10 (ICD-10) coded diagnoses for individuals admitted to any Welsh hospital and Welsh residents treated in English hospitals. This includes all inpatient and day case activity and includes both spell and episode level data. After the patient is discharged, handwritten notes are transcribed at each hospital into ICD-10 codes. | All Welsh hospital admissions, and Welsh residents treated in English hospitals, between 01/04/1995 and 05/02/2024. |
| Welsh Longitudinal General Practice Dataset (WLGP)^7^ | The WLGP includes attendance and clinical information for all general practice interactions at included practices. It includes data on demographics, symptoms, diagnoses, prescriptions and referrals. It can be linked to other datasets via anonymised fields for individuals and GPs. An electronic health record for each patient is maintained at the general practice on a clinical information system, with most data entered during the consultation with a clinician. Test results from secondary care are transferred into the system electronically. The majority of clinicians use Read Code terminology, but there are no standard rules for recording in primary care. | 83% of GP practices in Wales. 86% of Welsh population. 01/01/2000 - 01/07/2021. |
| Welsh Results Reporting Service (WRRS)^8^ | The WRRS contains laboratory results from across all health boards in Wales. The service allows clinicians to access results regardless of where they were requested or tested, saving time, test duplication and improving patient safety. | All tests requested from primary and secondary care NHS Wales organisations processed and analysed in NHS Wales laboratories. 01/06/1992 - 29/06/2021. |
| Welsh Demographic Service Dataset (WDSD)^9^ | The WDSD is an administrative database that contains information on all individuals registered with a GP in Wales. It includes anonymised address information and practice registration history. It allows linkage between anonymised individuals and anonymised households, allowing grouping of individuals into households. | All individuals registered with a GP in Wales, between 01/01/1990 and 06/05/2024. |

## *Supplementary Table 3. Good data matching is generally considered to be anonymised linkage field (ALF) status code of 1, 4 or 39.*

| ALF Status Code | ALF Status Description | Included |
| --- | --- | --- |
| 1 | NHS Number passes check digit test | Yes |
| 2 | NHS Number derived through external linkage, i.e. CRN match on PEDW | No |
| 4 | Surname, First Name, Postcode, Date of Birth and Gender Code match exactly to WDS | Yes |
| 35 | Fuzzy Matching probability >= 0.5 & < 0.9 | No |
| 39 | Fuzzy Matching probability >= 0.9 | Yes |
| 99 | No match or Fuzzy Matching probability < 0.5 | No |

## Supplementary Table 4. ICD-10 codes for acute myocardial infarction and stroke to identify potential cases

| Code | Description | Condition |
| --- | --- | --- |
| I210 | Acute transmural myocardial infarction of anterior wall | Myocardial Infarction |
| I211 | Acute transmural myocardial infarction of inferior wall | Myocardial Infarction |
| I212 | Acute transmural myocardial infarction of other sites | Myocardial Infarction |
| I213 | Acute transmural myocardial infarction of unspecified site | Myocardial Infarction |
| I214 | Acute subendocardial myocardial infarction | Myocardial Infarction |
| I219 | Acute myocardial infarction unspecified | Myocardial Infarction |
| I220 | Subsequent myocardial infarction of anterior wall | Myocardial Infarction |
| I221 | Subsequent myocardial infarction of inferior wall | Myocardial Infarction |
| I228 | Subsequent myocardial infarction of other sites | Myocardial Infarction |
| I229 | Subsequent myocardial infarction of unspecified site | Myocardial Infarction |
|  |  |  |
| I600 | Subarachnoid haemorrhage from carotid siphon and bifurcation | STROKE |
| I601 | Subarachnoid haemorrhage from middle cerebral artery | STROKE |
| I602 | Subarachnoid haemorrhage from anterior communicating artery | STROKE |
| I603 | Subarachnoid haemorrhage from posterior communicating artery | STROKE |
| I604 | Subarachnoid haemorrhage from basilar artery | STROKE |
| I605 | Subarachnoid haemorrhage from vertebral artery | STROKE |
| I606 | Subarachnoid haemorrhage from other intracranial arteries | STROKE |
| I607 | Subarachnoid haemorrhage from intracranial artery unspec | STROKE |
| I608 | Other subarachnoid haemorrhage | STROKE |
| I609 | Subarachnoid haemorrhage unspecified | STROKE |
| I610 | Intracerebral haemorrhage in hemisphere subcortical | STROKE |
| I611 | Intracerebral haemorrhage in hemisphere cortical | STROKE |
| I612 | Intracerebral haemorrhage in hemisphere unspecified | STROKE |
| I613 | Intracerebral haemorrhage in brain stem | STROKE |
| I614 | Intracerebral haemorrhage in cerebellum | STROKE |
| I615 | Intracerebral haemorrhage intraventricular | STROKE |
| I616 | Intracerebral haemorrhage multiple localized | STROKE |
| I618 | Other intracerebral haemorrhage | STROKE |
| I619 | Intracerebral haemorrhage unspecified | STROKE |
| I629 | Intracranial haemorrhage (nontraumatic)unspecified | STROKE |
| I630 | Cerebral infarct due to thrombosis of precerebral arteries | STROKE |
| I631 | Cerebral infarction due to embolism of precerebral arteries | STROKE |
| I632 | Cereb infarct due unsp occlusion or stenos precerebrl arts | STROKE |
| I633 | Cerebral infarction due to thrombosis of cerebral arteries | STROKE |
| I634 | Cerebral infarction due to embolism of cerebral arteries | STROKE |
| I635 | Cerebrl infarct due unspec occlusion or stenos cerebrl arts | STROKE |
| I636 | Cerebral infarction due to cerebral venous thrombosis, nonpyogenic | STROKE |
| I638 | Other cerebral infarction | STROKE |
| I639 | Cerebral infarction unspecified | STROKE |
| I64X | Stroke not specified as haemorrhage or infarction | STROKE |

## Supplementary Table 5.Sensitivity and subgroup analyses.

To assess the robustness of our findings, we undertook several predefined sensitivity analyses:

| **Sensitivity analyses** | |
| --- | --- |
| **MI** | To obtain a more accurate date on onset, we linked an elevated troponin concentration in WRRS with the PEDW event, using the test date as the date of the MI. Elevated troponin concentrations were defined as test results with a specimen collection date between seven days prior to the episode start date and episode end date, and with troponin T values >14ng/L and troponin I values >40ng/L (corresponding to the 99^th^ centile). This analysis included only individuals with an elevated troponin result. |
|  | We repeated the previous sensitivity analysis, this time defining an elevated troponin concentration as greater than 10x 99^th^ centile |
| **Stroke** | Excluded codes for subarachnoid haemorrhage from our list of stroke ICD-10 codes. |
|  | Restricted stroke cases to those with an ICD-10 code for cerebral infarction and unspecified stroke. |
|  | For stroke, we added an additional 91-180-day risk period. |
|  |  |
| **Subgroup analyses** | |
| Maximum CRP concentration (0-99, 100-199, 200-299, 300+ mg/L recorded within seven days of the BSI. | |
| Bacterial organism (*Escherichia coli* vs *Staphylococcus aureus* – as these were the two commonest BSI pathogens and archetypal Gram-negative and Gram-positive organisms respectively). | |
| Gender (male vs female).* | |

* Data on gender were obtained from PEDW, where “Gender identity is a person's sense of identification with either the male or female sex, as manifested in appearance, behaviour, and other aspects of a person's life” as stated by the individual.^10^

## Supplementary Figure 1. Diagrammatic representation of observation time for an individual in the proposed Self-Controlled Case Series design


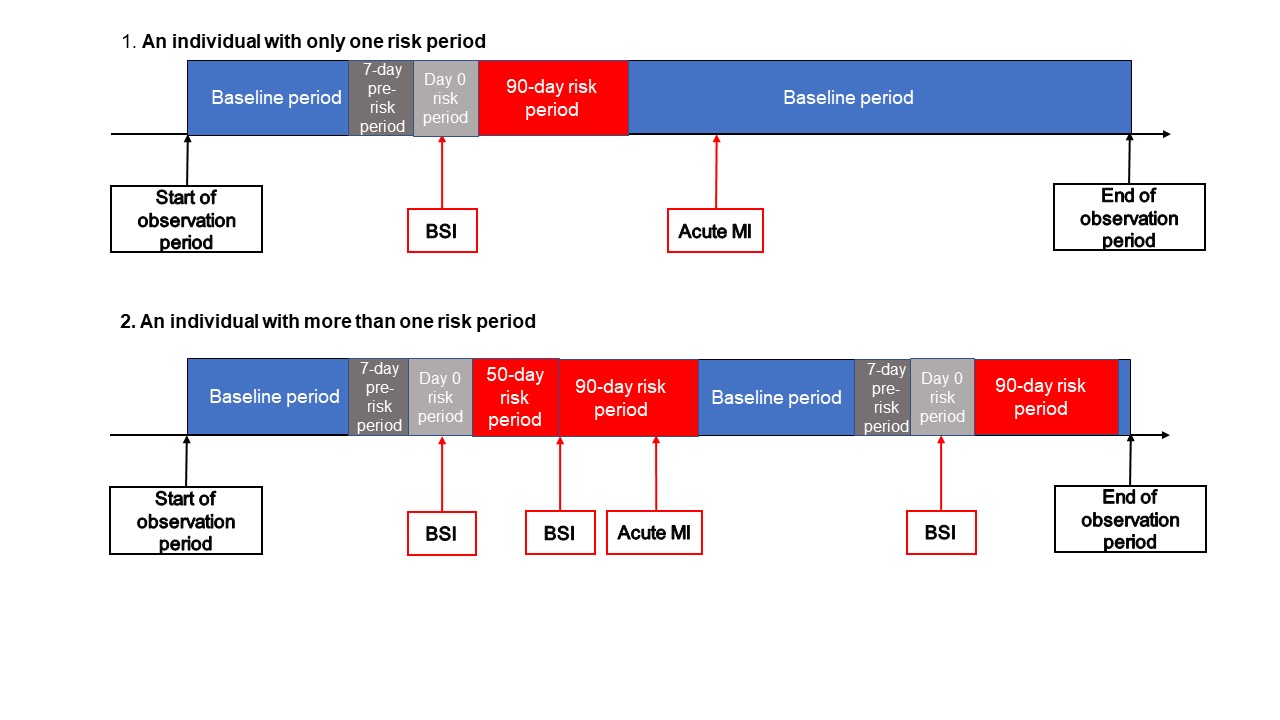


## Supplementary Figure 2 Selection of myocardial infarction (MI) and stroke cases.

*Individuals appear and are counted in both the stroke and MI datasets if they had both an MI and a stroke event within the study period.

SAIL = Secure Anonymised Information Linkage Databank; PEDW = Patient Episode Database for Wales; WDSD = Welsh Demographic Service Dataset; WLGP = Welsh Longitudinal General Practice data; ALF_STS_CD = Anonymised Linkage Field Status Code

All counts are rounded to the nearest 10.

## Supplementary Figure 3. Incidence rate ratios for myocardial infarction and stroke by organism.

## Supplementary Figure 4. Incidence rate ratios for myocardial infarction and stroke by peak C-reactive protein concentration.

## Supplementary Figure 5. Incidence rate ratios for myocardial infarction and stroke by aspirin and statin use.

References

1. Petersen I, Douglas I, Whitaker H. Self controlled case series methods: an alternative to standard epidemiological study designs. BMJ. 2016 Sep 12;i4515.

2. Gibson JE, Hubbard RB, Smith CJP, Tata LJ, Britton JR, Fogarty AW. Use of Self-controlled Analytical Techniques to Assess the Association Between Use of Prescription Medications and the Risk of Motor Vehicle Crashes. Am J Epidemiol. 2009 Jan 6;169(6):761–8.

3. Langan SM, Minassian C, Smeeth L, Thomas SL. Risk of Stroke Following Herpes Zoster: A Self-Controlled Case-Series Study. Clinical Infectious Diseases. 2014 Jun 1;58(11):1497–503.

4. Grave C, Boucheron P, Rudant J, Mikaeloff Y, Tubert-Bitter P, Escolano S, et al. Seasonal influenza vaccine and Guillain-Barré syndrome. Neurology. 2020 May 19;94(20).

5. Thomas SL, Minassian C, Ganesan V, Langan SM, Smeeth L. Chickenpox and Risk of Stroke: A Self-controlled Case Series Analysis. Clinical Infectious Diseases. 2014 Jan 1;58(1):61–8.

6. SAIL. Patient Episode Dataset for Wales (PEDW). Available from: https://web.www.healthdatagateway.org/dataset/ 4c33a5d2-164c-41d7-9797-dc2b008cc852. 2021.

7. SAIL. Welsh Longitudinal GP Dataset - Welsh Primary Care (WLGP). Available from: https://web.www.healthdatagateway.org/dataset/ 33fc3ffd-aa4c-4a16-a32f-0c900aaea3d2 . 2021.

8. SAIL. Welsh Results Reports Service (WRRS). Available from: https://web. www.healthdatagateway.org/dataset/71d37610-ac55-432d-82a3-bdb04407acd8. 2021.

9. SAIL. Welsh Demographic Service Dataset (WDSD). Available from: https://web.www.healthdatagateway.org/dataset/8a8a5e90-b0c6-4839-bcd2-c69e6e8dca6d. 2021.

10. Wales N. NHS Wales Data Dictionary: Gender. NHS Wales Data Dictionary, https://www.datadictionary.wales.nhs.uk/#!WordDocuments/gender.htm.
